# Imaging Anatomical Research on the Operative Windows of Oblique Lumbar Interbody Fusion

**DOI:** 10.1371/journal.pone.0163452

**Published:** 2016-09-29

**Authors:** Liehua Liu, Yong Liang, Hong Zhang, Haoming Wang, Congtao Guo, Xiaobing Pu, Chengmin Zhang, Liyuan Wang, Jian Wang, Yingwen Lv, Zhoukui Ren, Qiang Zhou, Zhongliang Deng

**Affiliations:** 1 Department of Orthopedics, Chongqing No. 13 People’s Hospital, Chongqing, China; 2 Department of Orthopedics, Orthopedics Center of PLA, Southwest Hospital, Third Military Medical University, Chongqing, China; 3 Department of Radiology, Southwest Hospital, Third Military Medical University, Chongqing, China; 4 Department 5, Chongqing Psychiatric Hospital, Chongqing, China; 5 Department of Orthopedics, 2nd Affiliated Hospital of Chongqing Medical University, Chongqing, China; Harvard Medical School/BIDMC, UNITED STATES

## Abstract

To provide applied anatomical evidence of the preoperative assessment of oblique lumbar interbody fusion (OLIF), the anatomical parameters of the OLIF operative window were observed through computed tomography angiography (CTA). We selected imaging data from 60 adults (30 males, 30 females) who underwent abdominal CTA and T12-S1 vertebral computed tomography (CT) with three-dimensional reconstruction. The OLIF operative windows at the L1-2, L2-3, L3-4, L4-5 and L5-S1 levels were as follows: the vascular window, bare window, psoas major window, ideal operative window, and actual operative window. Each level's actual operative window was statistically analyzed based on an actual operative window of <1 cm and ≥1 cm. The vascular window was largest at L4-5 (1.72 ± 0.58 cm). The bare window was largest at L5-S1 (1.59 ± 0.93 cm) and smallest at L3-4 (1.37 ± 0.51 cm). The psoas major window was largest at L3-4 (1.14 ± 0.35 cm) and smallest at L1-2 (0.41 ± 0.34 cm). The ideal operative window was largest at L4-5 (3.74 ± 0.36 cm) and smallest at L1-2 (3.23 ± 0.30 cm). The actual operative window was largest at L3-4, followed by L2-3, L4-5, L1-2, and L5-S1, which were 2.51 ± 0.56 cm, 2.28 ± 0.54 cm, 2.01 ± 0.74 cm, 1.80 ± 0.45 cm and 1.59 ± 0.93 cm, respectively (*P* = 0.000), and the percentages of the actual surgical window were 69%, 66%, 53%, 56% and 43%, respectively. The actual surgical window was <1 cm in 2 cases at L1-2 (3.3%), 4 cases at L4-5 (6.7%), and 17 cases at L5-S1 (28.3%) (11 males and 6 females). The regional anatomy of each level related to OLIF has its own peculiarities, and not all levels are suitable for OLIF. Before OLIF surgery, surgeons should analyze the imaging anatomy and select the appropriate surgical procedures.

## Introduction

In 2012, Silvestre *et al* reported on an oblique lumbar interbody fusion (OLIF) technique. [1] The patient was put in the right lateral decubitus position. A left abdominal oblique incision was selected, and the surgeon dissected through the muscle fibers of the obliquus externus abdominis, obliquus internus abdominis, and transversus abdominis into the extraperitoneal space. Blunt dissection was pointed to the place between the left psoas major and abdominal aorta in which the working channel was placed through which a lumbar discectomy and interbody fusion were performed. Because of the advantages such as the minimal surgical trauma, ease of operation, limited blood loss and quick recovery, additional spine surgeons have successively performed this surgery [2–4] and carried out related research. [5] Currently, the minimally invasive surgical (MIS) concept is widely preferred, and OLIF is likely to be a promising technique for lumbar interbody fusion.

The development of any new surgical technique is based on the local anatomical research. Because OLIF is an emerging technique, it lacks the systematic regional anatomical research necessary to guide surgeons to further develop the approach. Currently, imaging anatomical research related to OLIF has not been described in the literature. The authors believe that anatomical measurements made in vivo approximate the data and are both closer to the human surgical state [6] and more reliable.

Therefore, we observed the applied physiological anatomical parameters of OLIF operative windows through computed tomography angiography (CTA) and analyzed their clinical significance, thereby providing anatomical evidence for OLIF assessment.

## Materials and Methods

### General data

From August to October 2015, we selected imaging data from 60 adults who underwent abdominal CTA and T12-S1 vertebral computed tomography (CT) with three-dimensional reconstruction in our hospital. The patient sample included 30 males and 30 females, aged 42.5 ± 10.2 (19–68) years. None of the patients had lumbar anterior great vascular abnormalities or disorders, lumbar vertebra deformation, or a history of lumbar surgery or retroperitoneal surgery. The research has been approved by the Medical Ethics Committee of Chongqing No. 13 People’s Hospital. All subjects agreed to participate in this study and signed a written informed consent form.

### Methods

**Fig 1:**
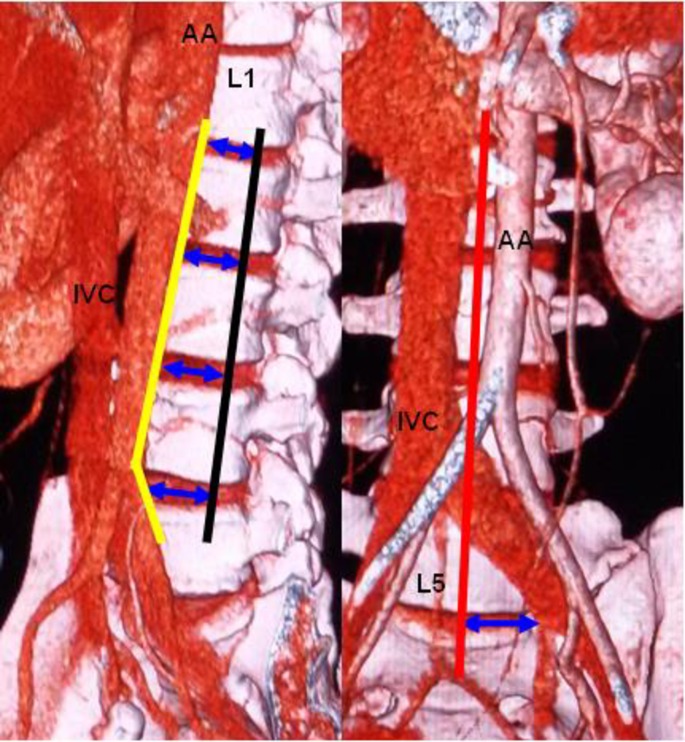
Each level's operative window. Yellow line: left boundary of the abdominal aorta or left iliac vessels; black line: middle frontal plane of the intervertebral space; red line: median sagittal plane; blue arrow: actual operative window; AA: abdominal aorta; IVC: inferior vena cava.

**Fig 2:**
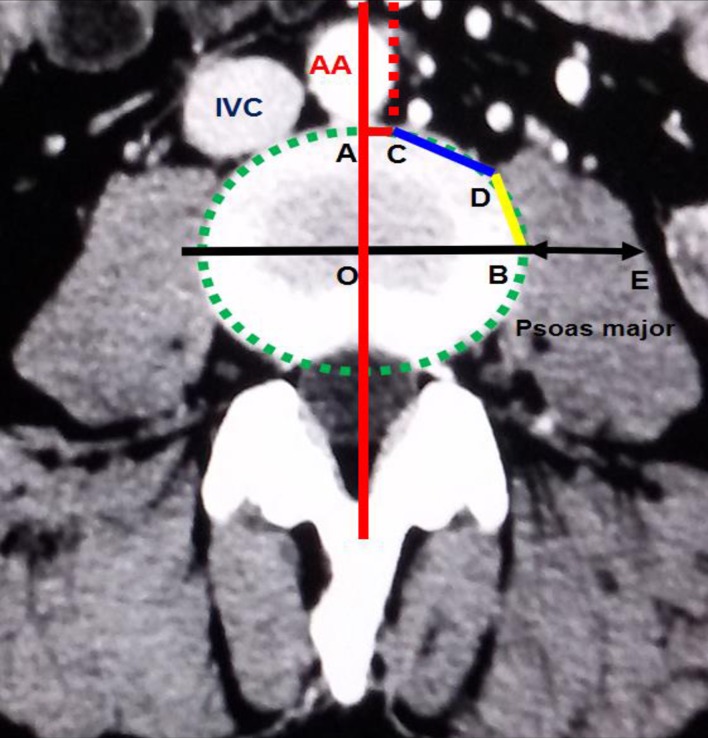
Transverse section of the L3-4 level. O: approximate oval intervertebral space center; AC: vascular window; CD: bare window; BD: psoas major window; BE: the left psoas major's width in the middle frontal plane; ideal operative window = vascular window (AC) + bare window (CD) + psoas major window (BD); actual operative window = bare window (CD) + psoas major window (BD).

**Fig 3:**
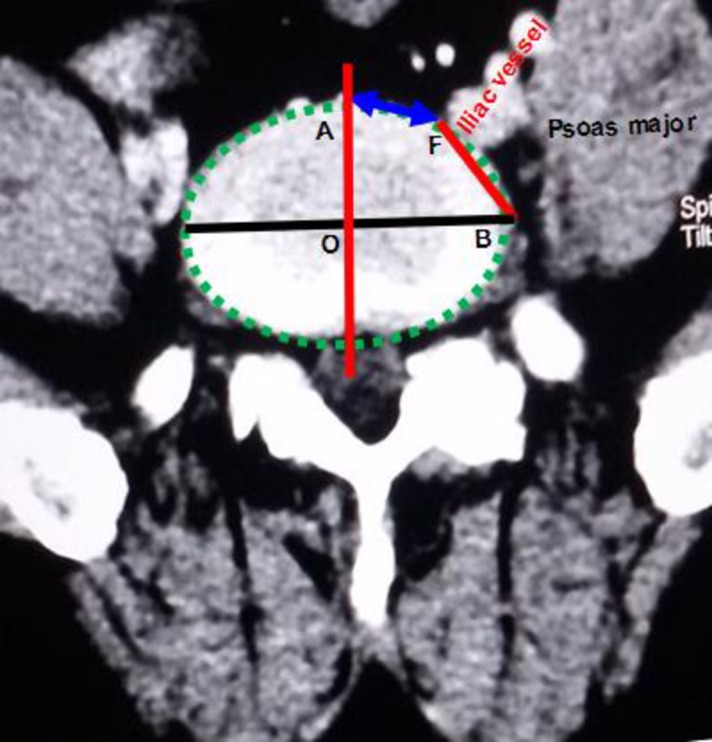
Transverse section of the L5-S1 level. AF: bare window = actual operative window.

A Somatom Definition dual-source spiral CT (SIEMENS Corporation) scanner was used for the T12-S1 vertebra and abdominal CTA. The scanner's parameters were as follows: slice thickness, 5 mm; pitch, 1.15 mm; reconstructive slice thickness, 1 mm; and overlapping rate, 30%. The contrast agent (Omnipaque 100 ml, 35 gI) was intravenously injected into the median cubital vein at a speed of 4–5 ml/s. The scan program was automatically triggered by a high-pressure syringe. The scanning time was 25–30 s in the arterial phase and 60–70 s in the venous phase. Using maximum intensity projection, volume rendering techniques, and multiplanar reformation, all images were clearly displayed. The post-processing workstation of the SIEMENS dual-source spiral CT was used to observe the OLIF operative windows at L1-2, L2-3, L3-4, L4-5 and L5-S1 (Fig 1). (1) The anatomical parameters of L1-2, L2-3, L3-4 and L4-5 (Fig 2) were as follows: the vascular window (the distance from the left boundary of the abdominal aorta or left iliac vessels to the median sagittal plane), the bare window (the left front of the intervertebral disc that is not covered by the abdominal aorta and left psoas major), the psoas major window (the left front of the intervertebral disc that is covered by the left psoas major) and the width of the left psoas major on the middle frontal plane of the intervertebral space. The ideal operative window = vascular window + bare window + psoas major window; and the actual operative window = bare window + psoas major window. (2) The percentages of the operative windows at L1-2, L2-3, L3-4 and L4-5 were calculated as follows: the percentage of the vascular window = vascular window ÷ ideal operative window × 100%; the percentage of the bare window = bare window ÷ ideal operative window × 100%; the percentage of the psoas major windows = psoas major windows ÷ ideal operative window × 100%; and the percentage of the actual operative window = actual operative window ÷ ideal operative window × 100%. (3) Because of the abdominal aortic bifurcation and common iliac venous confluence in front of the L5-S1 level, the operative window at L5-S1 had slightly different specification (Fig 3). The L5-S1 level had no psoas major window because the left psoas major did not cover the L5-S1 level. The left border of the bare window is the right boundary of the left iliac vessel, and the right border is the median sagittal plane. The bare window is equal to the actual operative window. The ideal operative window = bare window + BF (Fig 3). The percentage of the actual operative window = bare window (AF) ÷ ideal operative window. (4) Each level's actual operative window was statistically analyzed based on an actual operative window of <1 cm and ≥1 cm. (5) Additionally, we observed the positions of the renal artery and renal vein in front of the spine.

### Statistical analysis

SPSS19 software was employed for the statistical analysis. The anatomical parameters are presented as $\bar{x}$ ± S. An unpaired *t* test was used to analyze the differences between the males and females, and *P* < 0.05 indicated a significant difference. One-way analysis of variance was used to analyze the differences among all levels, and *P* < 0.05 indicated a significant difference.

## Results

The parameters of each level's operative window are listed in Tables 1 and 2. The left psoas major's width in the middle frontal plane is shown in Table 3. The statistics of each level's actual operative window according to its size are shown in Table 4. The walking planes of the renal artery and renal vein in front of the spine are listed in Tables 5 and 6.

**Table 1:** Parameters of the operative window for each level ($\bar{x}$ ± S, cm).

Operation window		Gender	L1-2	L2-3	L3-4	L4-5	L5-S1
Vascular window		Male	1.56 ± 0.48 (0.71–2.55)	1.24 ± 0.49 (0.00–1.93)	1.12 ± 0.48 (0.00–1.75)	1.49 ± 0.61 (0.00–2.56)	
		Female	1.32 ± 0.28 (0.60–1.85)	1.13 ± 0.38 (0.00–1.78)	1.14 ± 0.32 (0.52–1.84)	1.95 ± 0.46 (0.48–2.62)	
		*P*	0.023	0.344	0.817	0.002	
Actual operation window	Bare window	Male	1.48 ± 0.75 (0.50–3.18)	1.45 ± 0.67(0.56–3.44)	1.45 ± 0.66 (0.37–3.36)	1.58 ± 0.62(0.11–3.04)	1.36 ± 1.08 (0.00–3.02)
		Female	1.31 ± 0.39 (0.65–2.41)	1.39 ± 0.47 (0.42–2.74)	1.29 ± 0.29 (0.69–2.14)	1.31 ± 0.52 (0.41–2.40)	1.81 ± 0.71 (0.00–2.91)
		*P*	0.261	0.694	0.234	0.078	0.066
	Psoas major window	Male	0.43 ± 0.38 (0.00–1.00)	1.03 ± 0.28 (0.36–1.64)	1.35 ± 0.34 (0.82–1.89)	0.94 ± 0.47 (0.00–1.89)	
		Female	0.39 ± 0.29 (0.00–1.10)	0.69 ± 0.19 (0.32–0.99)	0.93 ± 0.20 (0.50–1.37)	0.20 ± 0.35 (0.00–1.07)	
		*P*	0.646	0.000	0.000	0.000	
Ideal operation window		Male	3.47 ± 0.23 (3.06–4.01)	3.72 ± 0.24 (3.26–4.16)	3.92 ± 0.26 (3.54–4.53)	4.01 ± 0.27 (3.70–4.65)	3.98 ± 0.33 (3.27–4.60)
		Female	3.02 ± 0.16 (2.69–3.25)	3.21 ± 0.19 (2.81–3.62)	3.36 ± 0.16 (3.08–3.66)	3.46 ± 0.18 (3.05–3.76)	3.39 ± 0.21 (2.98–3.82)
		*P*	0.000	0.000	0.000	0.000	0.000

**Table 2:** Parameters of the operative window for each level ($\bar{x}$ ± S, cm).

Operation window	L1-2	L2-3	L3-4	L4-5	L5-S1	*P*
Vascular window	1.44 ± 0.41 (0.60–2.55)	1.19 ± 0.44 (0.00–1.93)	1.13 ± 0.40 (0.00–1.84)	1.72 ± 0.58 (0.00–2.62)		0.017
Bare window	1.39 ± 0.60 (0.50–3.18)	1.42 ± 0.58 (0.42–3.44)	1.37 ± 0.51 (0.37–3.36)	1.44±0.59 (0.11–3.04)	1.59 ± 0.93 (0.00–3.02)	0.902
Psoas major window	0.41 ± 0.34 (0.00–1.10)	0.86 ± 0.29 (0.32–1.64)	1.14 ± 0.35 (0.50–1.89)	0.57 ± 0.55 (0.00–1.89)		0.000
Ideal operation window	3.23 ± 0.30 (2.69–4.61)	3.47 ± 0.33 (2.81–4.16	3.64 ± 0.35 (3.08–4.53)	3.74 ± 0.36 (3.05–4.65)	3.68 ± 0.40 (2.98–4.60)	0.097
Actual operation window	1.80 ± 0.45 (0.76–3.18)	2.28 ± 0.54 (1.40–3.80)	2.51 ± 0.56 (1.58–4.18)	2.01 ± 0.74 (0.68–3.62)	1.59 ± 0.93 (0.00–3.02)	0.000*

* Differences at all levels: *P*_L1-2 vs L2-3, L1-2 vs L3-4, L2-3 vs L4-5, L2-3 vs L5-S1, L3-4 vs L4-5, L3-4 vs L5-S1, L4-5 vs L5-S1_ < 0.05; *P*_L1-2 vs L4-5, L1-2 vs L5-S1,L2-3 vs L3-4_ > 0.05.

**Table 3:** Width of the left psoas major in the middle frontal plane ($\bar{x}$ ± S, cm).

Psoas major width	L1-2	L2-3	L3-4	L4-5	
Male	0.53 ± 0.47 (0.00–1.56)	1.46 ± 0.51 (0.81–2.50)	2.32 ± 0.63 (1.21–3.59)	3.46 ± 0.64 (2.55–4.63)	
Female	0.33 ± 0.23 (0.00–0.92)	0.92 ± 0.25 (0.34–1.42)	1.51 ± 0.32 (0.91–2.36)	2.58 ± 0.60 (0.00–3.41)	
*P*	0.048	0.000	0.000	0.000	
Male + female	0.43 ± 0.38 (0.00–1.56)	1.19 ± 0.49 (0.34–2.50)	1.92 ± 0.64 (0.91–3.59)	3.02 ± 0.76 (0.00–4.63)	*P* = 0.000

**Table 4:** Summary statistics of the actual operative window according to size for each level ($\bar{x}$ ± S, cm).

Actual operative Window		L1-2	L2-3	L3-4	L4-5	L5-S1
<1 cm	Male	0.85 ± 0.12 (2*) (0.76–0.93)				0.17 ± 0.64 (11*) (0.00–0.89)
	Female				0.84 ± 0.11 (4*) (0.68–0.93)	0.66 ± 0.33 (6*) (0.00–0.89)
	*P*					0.000
≥1 cm	Male	1.98 ± 0.49 (28*) (1.26–3.18)	2.48 ± 0.56 (30*) (1.58–3.80)	2.80 ± 0.60 (30*) (1.87–4.18)	2.51 ± 0.61 (30*) (1.40–3.62)	2.06 ± 0.52 (19*) (1.04–3.02)
	Female	1.69 ± 0.27 (30*) (1.21–2.41)	2.08 ± 0.44 (30*) (1.40–3.48)	2.22 ± 0.33 (30*) (1.58–2.90)	1.62 ± 0.42 (26*) (1.06–2.98)	2.09 ± 0.43 (24*) (1.28–2.91)
	*P*	0.008	0.003	0.000	0.000	0.800

* Note:The number of samples.

**Table 5:** The walking plane of the renal artery and renal vein in front of the spine.

Walking plane	Renal artery			Renal vein		
	Male (n = 30)	Female (n = 30)	Male+Female	Male (n = 30)	Female(n = 30)	Male+Female
T12 down 1/3					1 (3.3%)	1 (1.7%)
L1 up 1/3		4 13.3%)	4 (6.7%)	1 (3.3%)	1 (3.3%)	2 (3.3%)
L1 middle 1/3	7 (23.3%)	5 (16.7%)	12 (20.0%)	1 (3.3%)	3 (10.0%)	4 (6.7%)
L1 down 1/3	6 (20.0%)	5 (16.7%)	11 (18.3%)	7 (23.3%)	7 (23.3%)	14 (23.3%)
L1/2 intervertebral space*	5 (16.7%)	11 (36.7%)	16 (26.7%)	6 (20.0%)	10 (33.3%)	16 (26.7%)
L2 up 1/3	8 (26.7%)	4 (13.3%)	12 (20.0%)	9 (30.0%)	6 (20.0%)	15 (25.0%)
L2 middle 1/3	3 (10.0%)	1 (3.3%)	4 (6.7%)	5 (16.7%)	2 (6.7%)	7 (11.7%)
L2/3 intervertebral space	1 (3.3%)		1 (1.7%)	1 (3.3%)		1 (1.7%)

* The renal artery and renal vein were overlapping in front of the L1/2 intervertebral space in 11 cases (18.3%).

**Table 6:** Renal artery and renal vein positioning.

	Male (n = 30)	Female (n = 30)	Male+Female
Renal artery overlapping renal vein	16	15	31 (51.7%)
Renal artery above, renal vein below	9	11	20 (33.3%)
Renal vein above, renal artery below	5	4	9 (15.0%)

The vascular window was largest at L4-5 (1.72 ± 0.58 cm), followed by L1-2, L2-3 and L3-4, and their sizes significantly differed (*P* = 0.017). The differences at L4-5 and L1-2 between males and females were significant (*P* < 0.05). The bare window was largest at L5-S1 (1.59 ± 0.93 cm) and smallest at L3-4 (1.37 ± 0.51 cm), but the sizes did not significantly differ (*P* = 0.902) at all levels. Each level's bare window size did not significantly differ between males and females (*P* > 0.05). The psoas major window was largest at L3-4 (1.14 ± 0.35 cm) and smallest at L1-2 (0.41 ± 0.34 cm). At all levels, the psoas major window significantly differed (*P* = 0.000). The psoas major did not cover the L5-S1 level. The ideal operative window was largest at L4-5 and the smallest at L1-2, which were 3.74 ± 0.36 cm and 3.23 ± 0.30 cm, respectively, and there were no significant differences at all levels (*P* = 0.097). There were significant differences between males and females at all levels (*P* = 0.000). At the L4-5 level's middle frontal plane, the left psoas major's width was the widest (3.02 ± 0.76 cm), followed by L3-4, L2-3 and L1-2. The width of the left psoas major at the four levels significantly differed (*P* = 0.000), and the differences between males and females were significant at all levels (*P* < 0.05).

The following results are shown in Table 2. At L1-2, L2-3, L3-4 and L4-5, the vascular window accounted for 45%, 34%, 31% and 47% of the ideal operative window, respectively; the psoas major window accounted for 13%, 25%, 31% and 15% of the ideal window, respectively; and the bare window accounted for 42%, 41%, 38% and 39% of the ideal operative window, respectively. The L5-S1 level's bare window accounted for 43% of the ideal window.

The actual operative window was largest at L3-4, followed by L2-3, L4-5, L1-2 and L5-S1, which were 2.51 ± 0.56 cm, 2.28 ± 0.54 cm, 2.01 ± 0.74 cm, 1.80 ± 0.45 cm, and 1.59 ± 0.93 cm, respectively. At all levels, the actual operative window was significantly different (*P* = 0.000). At the L1-2, L2-3, L3-4, L4-5 and L5-S1 levels, the percentages of the actual surgical window were 56%, 66%, 69%, 53% and 43%, respectively.

At the L1-2, L2-3, L3-4 and L4-5 levels, the actual operative windows that were greater than 1 cm significantly differed between males and females (*P* < 0.05), with the windows being larger among males compared to females. At L5-S1, the actual operative windows more than 1 cm did not differ between males and females (*P* = 0.800), and the females’ windows were slightly larger than the males’ windows. The actual surgical windows were not more than 1 cm in 2 cases (3.3%) at L1-2, 4 cases (6.7%) at L4-5 and 17 cases (28.3%) at L5-S1 (11 males and 6 females).

Most of the renal artery walking planes were at the middle 1/3 of L1, down 1/3 of L1 and at the intervertebral space at L1/2. Most renal vein walking planes were 1/3 down L1, at the L1-2 intervertebral space and 1/3 up L2. The renal artery and renal vein were overlapping in front of the L1/2 intervertebral space in 11 cases (18.3%). The renal artery and renal vein were positioned in front of the L2/3 intervertebral space in 1 case. There were 31 cases (51.7%) of the renal artery and renal vein overlapping, including 11 cases (18.3%) with an overlap in front of the L1/2 intervertebral space, 20 cases (33.3%) in which the renal artery was above the renal vein, and 9 cases (15.0%) in which the renal artery was below the renal vein.

## Discussion

According to various surgical approaches, lumbar interbody fusion is divided into fusion styles including anterior lumbar interbody fusion (ALIF), posterior lumbar interbody fusion (PLIF), transforaminal lumbar interbody fusion (TLIF) and extreme lateral lumbar interbody fusion (XLIF). In 1932, Capener first reported that he had treated spondylolisthesis with ALIF, which aimed to restore the height of the intervertebral space and expand the intervertebral foramen. In recent years, with the continuous development of anterior lumbar fixation devices, [7] ALIF has been extensively developed. However, ALIF involves complications such as abdominal visceral injury, anterior lumbar vascular injury, retrograde ejaculation, intestinal adhesion and abdominal hernia. [8–9] A recent study reported that retrograde ejaculation occurred in 7.4% of cases, [10] and vascular injury occurred in 6.1% of cases. [11] Laparoscopic ALIF is not suitable for wide use because of its complex operation, many procedures, limited field of vision and long learning curve. [12]

As representatives of posterior lumbar fusion, PLIF and TLIF require extensive stripping of the paraspinal muscles, with an extensive exposure range, intense trauma, substantial bleeding and severe damage to the lumbar biomechanical structure and function, resulting in complications such as slow postoperative recovery, back stiffness, and chronic back pain. In particular, PLIF easily leads to dural laceration and nerve root tractive injury. In recent years, minimally invasive posterior lumbar fusion included the Wiltse approach, but the muscles, ligaments and tendon insertions were damaged, and exposure is limited, increasing the difficulty of the surgery. In 2006, Ozgur *et al* described an XLIF [13] and completed the operation using a working channel through the psoas major. This approach has some advantages, including minimal invasiveness, less bleeding, maximum expansion of the spinal canal and nerve compression relief with a larger interbody fusion cage placed in the disc space. [14] Because the psoas major contains important nerves that extend to the lower limbs, intraoperative neurophysiological monitoring is required but is complicated [15]. The main postoperative complications were thigh numbness or pain and reduced lower-limb muscle strength. There was a study that reported that the incidence rates of sensory disturbances and dyskinesia were increased to 75% and 33.6%, respectively. [16]

Based on these complications with ALIF, PLIF and XLIF, OLIF is the new surgical lumbar fusion technique used in recent years. The main advantages of OLIF are as follows: (1) Through the bare window between the abdominal aorta and the left psoas major, the working channel is placed to complete the operation, which is minimally invasive, causes less bleeding, and requires a shorter operative time. Its incision is a sliding window, which can be completed by fusing 3 levels at most. [1] (2) A larger area and volume of the interbody fusion device can be imbedded, which can effectively restore the disc space and intervertebral foramen height, thus allowing indirect spinal canal decompression. [3, 17, 18] (3) The approach is not through the peritoneal cavity and thus avoids interfering with the abdominal organs, and the incision in the abdominal wall is along the muscle fibers. This technique maintains the dominance of the rectus abdominis and reduces nerve damage, improves postoperative wound healing, and prevents abdominal hernia. [5] (4) The left psoas major only needs to be appropriately stripped, and intraoperative neurophysiological monitoring is not needed. [5]

Fujibayashi *et al* [2] performed a study with 28 patients with lumbar stenosis who had undergone oblique lateral interbody fusion combined with percutaneous pedicle screw fixation at 52 lumbar levels. All of the cases' clinical symptoms improved without neurological complications. The mean cross-sectional area of the thecal sac increased from 99.6 mm preoperatively to 134.3 mm postoperatively. Silvestre *et al* [1] completed 179 OLIFs, with 1 level in 56, 2 levels in 107, and 3 levels in 16 patients. The procedure was associated with minimal blood loss (57 ± 131 ml) and a short operation time (32.5 ± 13.2 min). Complications included incision pain in 4 cases, sympathetic chain injury in 3 cases, neurological deficit in 2 cases and iliac vein laceration in 3 cases. Ohtori S *et al* [17, 18] completed 35 OLIFs for lumbar spinal degeneration disease and 12 OLIFs for degenerated lumbar spinal kyphoscoliosis, which had good surgical results without major complications. Kanno *et al* [3] also reported on two cases of L5-S1 OLIF with better clinical results. Wakita *et al* [4] successfully combined OLIF with percutaneous pedicle screw fixation to treat one lumbar kyphoscoliosis case caused by an L4 lumbar fracture.

Silvestre *et al* [1] reported a total of 20 cases (11.2%) of surgical complications, including iliac vascular complications in 3 cases (1.8%), and one L5-S1 OLIF case that had to be aborted and switched to another operation. Thus, we should pay great attention to individual differences in the OLIF regional anatomy, particularly in the preoperative analysis. Davis *et al* [5] conducted an autopsy study related to OLIF, with findings of important significance. He measured the access corridor diameters in 20 cadavers in the static state and with mild psoas major retraction, which were as follows: 18.60 mm and 25.50 mm at L2-3; 19.25 mm and 27.05 mm at L3-4; 15.00 mm and 24.45 mm at L4-5; and 14.75 mm at L5-S1. The two anatomical parameters of each segment in his study were similar to our imaging parameters of the bare window and the actual surgical window. The parameters are slightly higher than those observed in the present study, possibly because the cadavers were soaked in formalin and had undergone protein coagulation and tissue retraction. In addition, in general, American bodies are taller and larger than Asian bodies. We measured the individual data in vivo, which more closely represents the surgical state.

In OLIF surgery, to increase exposure and possibly place the highest and longest cage in the median coronal plane of the intervertebral space, the surgeon needs to strip a part of the left psoas major that covers the intervertebral space and tract the left psoas major to the left rear. [5] Therefore, we observed the vascular window, bare window, and psoas major window, which are closely related to the OLIF site. The vascular window is a surgically restricted area and is obstructed by the abdominal aorta or left iliac vessel at L1-2, L2-3, L3-4, and L4-5. The bare window is exposed and can be a direct surgical area. The bare window and psoas major window together constitute the actual operative window of the OLIF site. The actual operative window is larger and the exposure of the surgical field, the indirect decompression of the canal and intervertebral fusion are performed more easily, thus potentially resulting in fewer surgical complications. The size of the psoas major window is the stripped psoas major scope, which increases the surgical area. However, when we strip the left psoas major, we do not exceed the middle frontal plane because the middle frontal plane has reached the left border of the intervertebral disc. Furthermore, significant stripping of the psoas major may stimulate the lumbar plexus.

**Fig 4:**
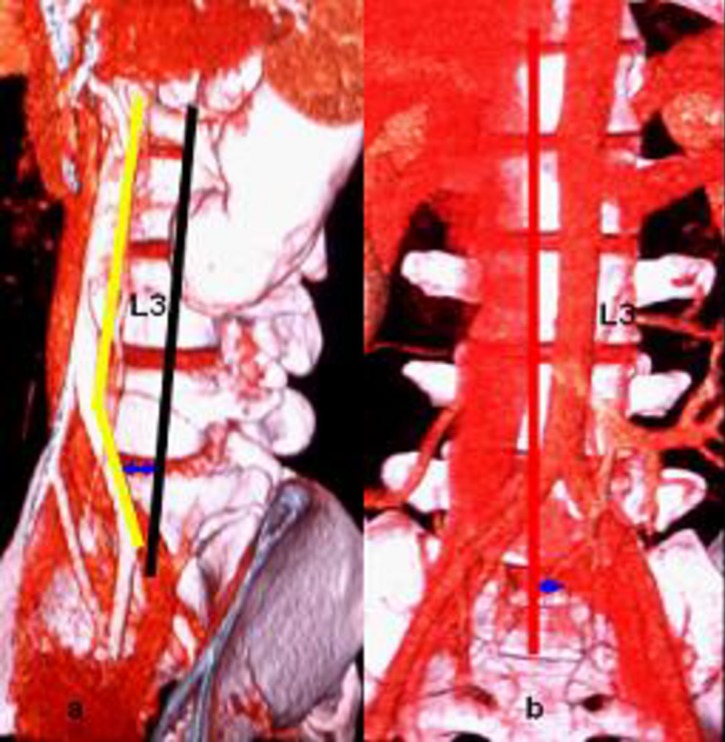
a: Case 1: The L4-5 OLIF actual operative window (blue arrow) is 0.93 cm; b: case 2: The L5-S1 OLIF actual operative window (blue arrow) is 0.52 cm.

The width of the cage used for PLIF is 8–11 mm, and the minimal width of the cross-fuse used for XLIF is 14 mm. Although the cage used for OLIF has no uniform standard, its width should be at least 1 cm. The actual operative window for OLIF is not less than the width of a cage, so we studied each level's actual operative window using the thresholds of ≥1.00 cm and <1.00 cm. At L3-4 and L2-3, the actual operative windows were more than 1 cm, accounting for approximately two-thirds of the ideal operative window (L3-4 69%, L2-3 66%), which easily allows for the completion of the surgical procedure. At the L1-2, L4-5, and L5-S1 levels, 3.3%, 6.7%, and 28.3% of the population have actual operative windows less than 1 cm, respectively (Fig 4). Therefore, OLIF is not suitable for all people. Those who were not suitable candidates for OLIF at the L1-2 level were men and at the L4-5 level, women. At the L5-S1 level, 18.3% of males and 10.0% of females are not suitable for OLIF. Because abdominal aortic bifurcation and iliac vein confluence focused on the front of L4-L5 and the OLIF's operative window bordered the iliac vein, it should be noted that the risk of vascular complications is higher at the L4-5 and L5-S1 levels. [1, 3] The authors believe that before OLIF surgery, an imaging examination of the great vessels in front of the lumbar region should be performed [6]. If the actual operative window is less than 1 cm, the surgeon should switch to other lumbar fusion techniques.

OLIF requires implanting a larger model cage to indirectly expand the spinal canal and intervertebral foramen and restore the lumbar mechanical stability in the coronal and sagittal planes. [3] Therefore, the cage should be implanted in the intervertebral space in the median coronal plane. The left psoas major's width in the middle frontal plane of the L4-5 intervertebral space is largest, followed by L3-4, L2-3, and L1-2. To place the cage in a satisfactory position in L4-5 OLIF, the left psoas major requires a larger force to retract on the left rear, reducing the barrier that comes from the psoas major when the cage is hammered.

**Fig 5:**
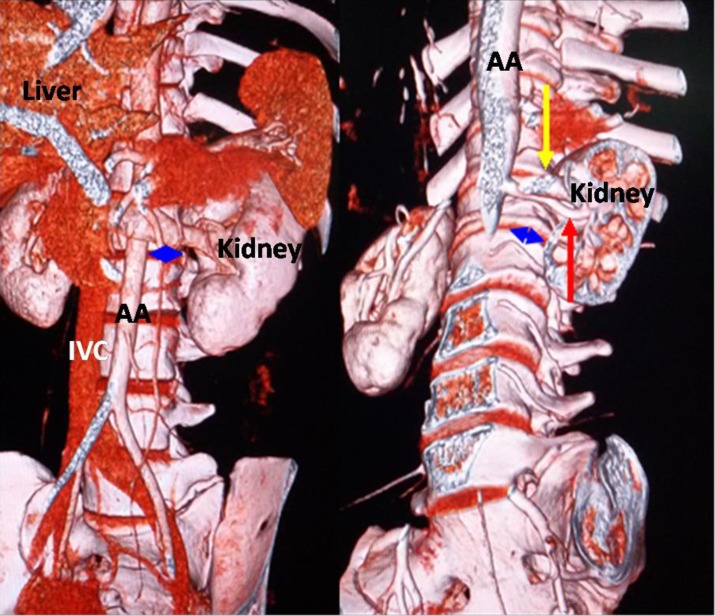
The renal artery and renal vein were overlapping and positioned anteriorly 1/3 down L1. Red arrow: renal artery; yellow arrow: renal vein; blue arrow: the actual operative window of the L1-2 level.

Silvestre *et al* [1] reported that OLIF had been completed at the L1-2 level. However, at present, the anatomical research of the L1-2 level related to OLIF has not been described. We observed the operative window at the L1-2 level, as well as the positions of the renal artery and renal vein in front of the spine. At the L1-2 level, the working channel was behind the renal artery and renal vein and was adjacent to the renal vasculature, which is located anteriorly down 1/3 of L1, at the L1-2 disc space, and 1/3 up L2 (Fig 5). Caution is necessary to prevent renal vascular injury. With the gradual increase in L1-2 OLIF use, renal vascular injury may be a noteworthy complication. The renal vasculature may be located anteriorly at the L2-3 level, so surgeons need to be vigilant when working in this location.

The study of the regional anatomy mainly includes autopsy research and imagology research. Autopsy research employs direct observation and measurement, but imagology research produces indirect measures with imaging equipment, which produces results that are closer to the physiological state. Currently, only one autopsy study related to OLIF has been reported, [5] so there are insufficient data from different research methods for intercomparisons and conclusions about which study is more advantageous and closer to the gold standard. The results of this study and autopsy research are similar, and their consequences are reliable. Of course, any study has its limitations, and the present study is no exception. First, there may be errors in the measurements of the operative window. Additionally, the actual operative window equals the bare window plus the psoas major window, not the straight-line distances between two points (C point and D point). Thus, the actual operative window is slightly larger than the actual values presented in this paper. Finally, the results of this anatomical imaging study should be combined with clinical information to further verify our conclusions, which will be the author's next research goal.

### Conclusion

The regional anatomy of each level related to OLIF has its own peculiarities, and not all levels are suitable for OLIF. At the L1-2, L4-5, and L5-S1 levels, 3.3%, 6.7%, and 28.3% of the population are not suitable for OLIF, respectively. Before OLIF surgery, surgeons should analyze the local anatomical characteristics using an imaging examination (CT, MRI or angiography) to screen patients and select appropriate surgical procedures.

## Supporting Information

S1 Fig Each level's operative window. Yellow line: left boundary of the abdominal aorta or left iliac vessels; black line: middle frontal plane of the intervertebral space; red line: median sagittal plane; blue arrow: actual operative window; AA: abdominal aorta; IVC: inferior vena cava.
(DOCX)

S2 Fig Transverse section of the L3-4 level. O: approximate oval intervertebral space center; AC: vascular window; CD: bare window; BD: psoas major window; BE: the left psoas major's width in the middle frontal plane; ideal operative window = vascular window (AC) + bare window (CD) + psoas major window (BD); actual operative window = bare window (CD) + psoas major window (BD).
(DOCX)

S3 Fig Transverse section of the L5-S1 level. AF: bare window = actual operative window.
(DOCX)

S4 Fig Actual operative window <1cm. a: Case 1: The L4-5 OLIF actual operative window (blue arrow) is 0.93 cm; b: case 2: The L5-S1 OLIF actual operative window (blue arrow) is 0.52 cm.
(DOCX)

S5 Fig The renal artery and renal vein were overlapping and positioned anteriorly 1/3 down L1. Red arrow: renal artery; yellow arrow: renal vein; blue arrow: the actual operative window of the L1-2 level.
(DOCX)

S1 Table Parameters of the operative window for each level. (DOCX)

S2 Table Parameters of the operative window for each level. (DOCX)

S3 Table Width of the left psoas major in the middle frontal plane. (DOCX)

S4 Table Summary statistics of the actual operative window according to size for each level. (DOCX)

S5 Table The walking plane of the renal artery and renal vein in front of the spine. (DOCX)

S6 Table Renal artery and renal vein positioning. (DOCX)
