# Supplementary material for: Imaging Anatomical Research on the Operative Windows of Oblique Lumbar Interbody Fusion
Source: PLoS One. 2016 Sep 29;11(9):e0163452. doi: 10.1371/journal.pone.0163452 (PMC5042505; doi:10.1371/journal.pone.0163452)
Supplement: S1 Fig — Yellow line: left boundary of the abdominal aorta or left iliac vessels; black line: middle frontal plane of the intervertebral space; red line: median sagittal plane; blue arrow: actual operative window; AA: abdominal aorta; IVC: inferior vena cava. (DOCX) [file pone.0163452.s001.docx]

**S1 Fig. Each level's operative window.** Yellow line: left boundary of the abdominal aorta or left iliac vessels; black line: middle frontal plane of the intervertebral space; red line: median sagittal plane; blue arrow: actual operative window; AA: abdominal aorta; IVC: inferior vena cava.

**
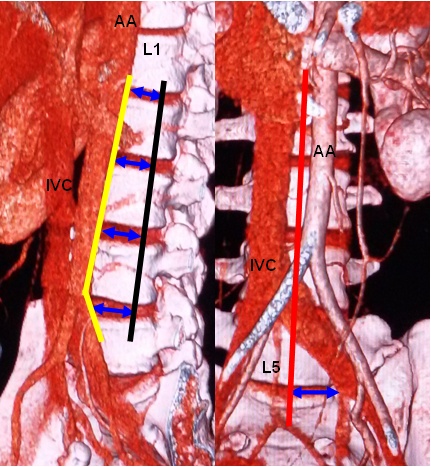
**
