# Supplementary material for: Imaging Anatomical Research on the Operative Windows of Oblique Lumbar Interbody Fusion
Source: PLoS One. 2016 Sep 29;11(9):e0163452. doi: 10.1371/journal.pone.0163452 (PMC5042505; doi:10.1371/journal.pone.0163452)
Supplement: S2 Fig — O: approximate oval intervertebral space center; AC: vascular window; CD: bare window; BD: psoas major window; BE: the left psoas major's width in the middle frontal plane; ideal operative window = vascular window (AC) + bare window (CD) + psoas major window (BD); actual operative window = bare window (CD) + psoas major window (BD). (DOCX) [file pone.0163452.s002.docx]

**S2 Fig. Transverse section of the L3-4 level.** O: approximate oval intervertebral space center; AC: vascular window; CD: bare window; BD: psoas major window; BE: the left psoas major's width in the middle frontal plane; ideal operative window = vascular window (AC) + bare window (CD) + psoas major window (BD); actual operative window = bare window (CD) + psoas major window (BD).

**
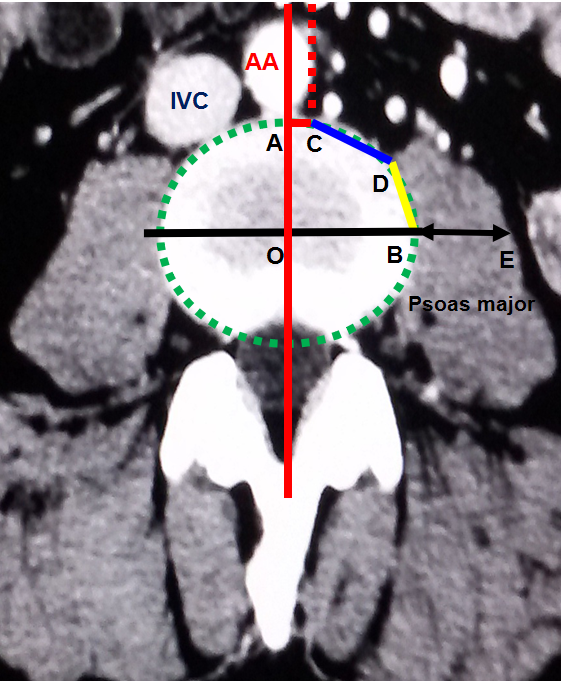
**
