# Supplementary figures and images for: Imaging Anatomical Research on the Operative Windows of Oblique Lumbar Interbody Fusion
Source: PLoS One. 2016 Sep 29;11(9):e0163452. doi: 10.1371/journal.pone.0163452 (PMC5042505; doi:10.1371/journal.pone.0163452)

**S3 Fig. Transverse section of the L5-S1 level.** AF: bare window = actual operative window


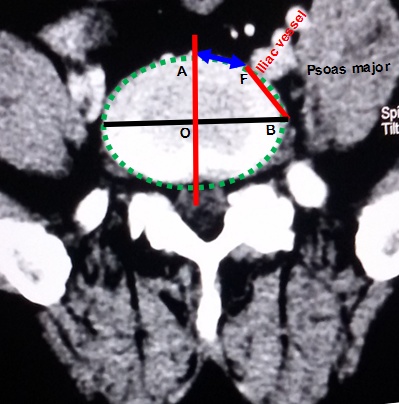

Supplement: S3 Fig — AF: bare window = actual operative window. (DOCX) [file pone.0163452.s003.docx]
