# Supplementary material for: Imaging Anatomical Research on the Operative Windows of Oblique Lumbar Interbody Fusion
Source: PLoS One. 2016 Sep 29;11(9):e0163452. doi: 10.1371/journal.pone.0163452 (PMC5042505; doi:10.1371/journal.pone.0163452)

**S4 Fig.** **Actual operative window ﹤1cm.** a: Case 1: The L4-5 OLIF actual operative window (blue arrow) is 0.93 cm; b: case 2: The L5-S1 OLIF actual operative window (blue arrow) is 0.52 cm.


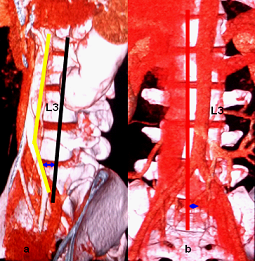

Supplement: S4 Fig — a: Case 1: The L4-5 OLIF actual operative window (blue arrow) is 0.93 cm; b: case 2: The L5-S1 OLIF actual operative window (blue arrow) is 0.52 cm. (DOCX) [file pone.0163452.s004.docx]
