# Supplementary material for: Imaging Anatomical Research on the Operative Windows of Oblique Lumbar Interbody Fusion
Source: PLoS One. 2016 Sep 29;11(9):e0163452. doi: 10.1371/journal.pone.0163452 (PMC5042505; doi:10.1371/journal.pone.0163452)

**S5 Fig. The renal artery and renal vein were overlapping and positioned anteriorly 1/3 down L1**. Red arrow: renal artery; yellow arrow: renal vein; blue arrow: the actual operative window of the L1-2 level.


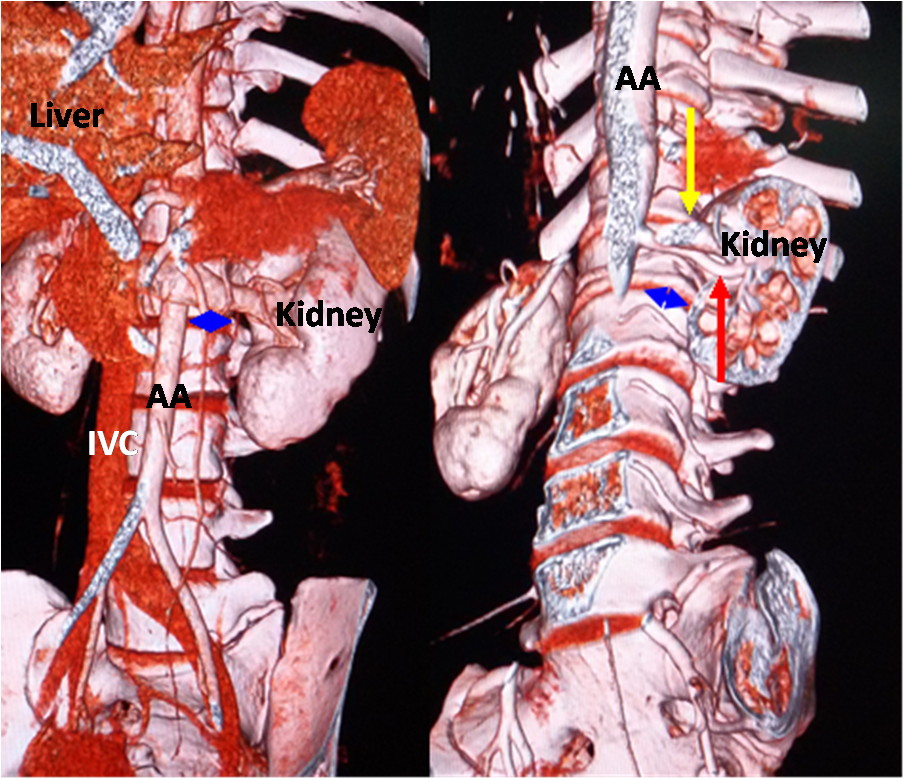

Supplement: S5 Fig — Red arrow: renal artery; yellow arrow: renal vein; blue arrow: the actual operative window of the L1-2 level. (DOCX) [file pone.0163452.s005.docx]
