# Supplementary material for: Imaging Anatomical Research on the Operative Windows of Oblique Lumbar Interbody Fusion
Source: PLoS One. 2016 Sep 29;11(9):e0163452. doi: 10.1371/journal.pone.0163452 (PMC5042505; doi:10.1371/journal.pone.0163452)
Supplement: S1 Table — (DOCX) [file pone.0163452.s006.docx]

**S1 Table. Parameters of the operative window for each level.**

Table 1. Parameters of the operative window for each level (‾x ± S, cm)

| Operation window | | Gender | L1-2 | L2-3 | L3-4 | L4-5 | L5-S1 |
| --- | --- | --- | --- | --- | --- | --- | --- |
| Vascular window | | Male | 1.56 ± 0.48 (0.71 - 2.55) | 1.24 ± 0.49 (0.00 - 1.93) | 1.12 ± 0.48 (0.00 - 1.75) | 1.49 ± 0.61 (0.00 - 2.56) |  |
|  |  | Female | 1.32 ± 0.28 (0.60 - 1.85) | 1.13 ± 0.38 (0.00 - 1.78) | 1.14 ± 0.32 (0.52 - 1.84) | 1.95 ± 0.46 (0.48 - 2.62) |  |
|  |  | *P* | 0.023 | 0.344 | 0.817 | 0.002 |  |
| Actual operation window | Bare window | Male | 1.48 ± 0.75 (0.50 - 3.18) | 1.45 ± 0.67(0.56 - 3.44) | 1.45 ± 0.66 (0.37 - 3.36) | 1.58 ± 0.62(0.11 - 3.04) | 1.36 ± 1.08 (0.00 - 3.02) |
|  |  | Female | 1.31 ± 0.39 (0.65 - 2.41) | 1.39 ± 0.47 (0.42 - 2.74) | 1.29 ± 0.29 (0.69 - 2.14) | 1.31 ± 0.52 (0.41 - 2.40) | 1.81 ± 0.71 (0.00 - 2.91) |
|  |  | *P* | 0.261 | 0.694 | 0.234 | 0.078 | 0.066 |
|  | Psoas major window | Male | 0.43 ± 0.38 (0.00 - 1.00) | 1.03 ± 0.28 (0.36 - 1.64) | 1.35 ± 0.34 (0.82 - 1.89) | 0.94 ± 0.47 (0.00 - 1.89) |  |
|  |  | Female | 0.39 ± 0.29 (0.00 - 1.10) | 0.69 ± 0.19 (0.32 - 0.99) | 0.93 ± 0.20 (0.50 - 1.37) | 0.20 ± 0.35 (0.00 - 1.07) |  |
|  |  | *P* | 0.646 | 0.000 | 0.000 | 0.000 |  |
| Ideal operation window | | Male | 3.47 ± 0.23 (3.06 - 4.01) | 3.72 ± 0.24 (3.26 - 4.16) | 3.92 ± 0.26 (3.54 - 4.53) | 4.01 ± 0.27 (3.70 - 4.65) | 3.98 ± 0.33 (3.27 - 4.60) |
|  |  | Female | 3.02 ± 0.16 (2.69 - 3.25) | 3.21 ± 0.19 (2.81 - 3.62) | 3.36 ± 0.16 (3.08 - 3.66) | 3.46 ± 0.18 (3.05 - 3.76) | 3.39 ± 0.21 (2.98 - 3.82) |
|  |  | *P* | 0.000 | 0.000 | 0.000 | 0.000 | 0.000 |
