# Supplementary material for: Imaging Anatomical Research on the Operative Windows of Oblique Lumbar Interbody Fusion
Source: PLoS One. 2016 Sep 29;11(9):e0163452. doi: 10.1371/journal.pone.0163452 (PMC5042505; doi:10.1371/journal.pone.0163452)
Supplement: S2 Table — (DOCX) [file pone.0163452.s007.docx]

**S2 Table. Parameters of the operative window for each level.**

Table 2. Parameters of the operative window for each level (‾x± S, cm)

| Operation window | L1-2 | L2-3 | L3-4 | L4-5 | L5-S1 | *P* |
| --- | --- | --- | --- | --- | --- | --- |
| Vascular window | 1.44 ± 0.41 (0.60 - 2.55) | 1.19 ± 0.44 (0.00 - 1.93) | 1.13 ± 0.40 (0.00 - 1.84) | 1.72 ± 0.58 (0.00 - 2.62) |  | 0.017 |
| Bare window | 1.39 ± 0.60 (0.50 - 3.18) | 1.42 ± 0.58 (0.42 - 3.44) | 1.37 ± 0.51 (0.37 - 3.36) | 1.44±0.59 (0.11 - 3.04) | 1.59 ± 0.93 (0.00 - 3.02) | 0.902 |
| Psoas major window | 0.41 ± 0.34 (0.00 - 1.10) | 0.86 ± 0.29 (0.32 - 1.64) | 1.14 ± 0.35 (0.50 - 1.89) | 0.57 ± 0.55 (0.00 - 1.89) |  | 0.000 |
| Ideal operation window | 3.23 ± 0.30 (2.69-4.61) | 3.47 ± 0.33 (2.81 - 4.16 | 3.64 ± 0.35 (3.08 - 4.53) | 3.74 ± 0.36 (3.05 - 4.65) | 3.68 ± 0.40 (2.98 - 4.60) | 0.097 |
| Actual operation window | 1.80 ± 0.45 (0.76 - 3.18) | 2.28 ± 0.54 (1.40 - 3.80) | 2.51 ± 0.56 (1.58 - 4.18) | 2.01 ± 0.74 (0.68 - 3.62) | 1.59 ± 0.93 (0.00 - 3.02) | 0.000^*^ |

^*^ Differences at all levels: *P* _L1-2 vs L2-3, L1-2 vs L3-4, L2-3 vs L4-5, L2-3 vs L5-S1, L3-4 vs L4-5, L3-4 vs L5-S1, L4-5 vs L5-S1_ < 0.05; *P* _L1-2 vs L4-5, L1-2 vs L5-S1,L2-3 vs L3-4_ ＞ 0.05.
