# Supplementary material for: Imaging Anatomical Research on the Operative Windows of Oblique Lumbar Interbody Fusion
Source: PLoS One. 2016 Sep 29;11(9):e0163452. doi: 10.1371/journal.pone.0163452 (PMC5042505; doi:10.1371/journal.pone.0163452)
Supplement: S3 Table — (DOCX) [file pone.0163452.s008.docx]

**S3 Table. Width of the left psoas major in the middle frontal plane.**

| Psoas major width | L1-2 | L2-3 | L3-4 | L4-5 |  |
| --- | --- | --- | --- | --- | --- |
| Male | 0.53 ± 0.47 (0.00 - 1.56) | 1.46 ± 0.51 (0.81 - 2.50) | 2.32 ± 0.63 (1.21 - 3.59) | 3.46 ± 0.64 (2.55 - 4.63) |  |
| Female | 0.33 ± 0.23 (0.00-0.92) | 0.92 ± 0.25 (0.34 - 1.42) | 1.51 ± 0.32 (0.91 - 2.36) | 2.58 ± 0.60 (0.00 - 3.41) |  |
| *P* | 0.048 | 0.000 | 0.000 | 0.000 |  |
| Male + female | 0.43 ± 0.38 (0.00 - 1.56) | 1.19 ± 0.49 (0.34 - 2.50) | 1.92 ± 0.64 (0.91 - 3.59) | 3.02 ± 0.76 (0.00 - 4.63) | *P* = 0.000 |
