# Supplementary material for: Imaging Anatomical Research on the Operative Windows of Oblique Lumbar Interbody Fusion
Source: PLoS One. 2016 Sep 29;11(9):e0163452. doi: 10.1371/journal.pone.0163452 (PMC5042505; doi:10.1371/journal.pone.0163452)
Supplement: S4 Table — (DOCX) [file pone.0163452.s009.docx]

**S4 Table. Summary statistics of the actual operative window according to size for each level.**

| Actual operative Window | | L1-2 | L2-3 | L3-4 | L4-5 | L5-S1 |
| --- | --- | --- | --- | --- | --- | --- |
| ＜1 cm | Male | 0.85 ± 0.12 (2^*^) (0.76 - 0.93) |  |  |  | 0.17 ± 0.64 (11^*^) (0.00 - 0.89) |
|  | Female |  |  |  | 0.84 ± 0.11 (4^*^) (0.68 - 0.93) | 0.66 ± 0.33 (6^*^) (0.00 - 0.89) |
|  | *P* |  |  |  |  | 0.000 |
| ≥1 cm | Male | 1.98 ± 0.49 (28^*^) (1.26 - 3.18) | 2.48 ± 0.56 (30^*^) (1.58 - 3.80) | 2.80 ± 0.60 (30^*^) (1.87 - 4.18) | 2.51 ± 0.61 (30^*^) (1.40 - 3.62) | 2.06 ± 0.52 (19^*^) (1.04 - 3.02) |
|  | Female | 1.69 ± 0.27 (30^*^) (1.21 - 2.41) | 2.08 ± 0.44 (30^*^) (1.40 - 3.48) | 2.22 ± 0.33 (30^*^) (1.58 - 2.90) | 1.62 ± 0.42 (26^*^) (1.06 - 2.98) | 2.09 ± 0.43 (24^*^) (1.28 - 2.91) |
|  | *P* | 0.008 | 0.003 | 0.000 | 0.000 | 0.800 |
