# Supplementary material for: Imaging Anatomical Research on the Operative Windows of Oblique Lumbar Interbody Fusion
Source: PLoS One. 2016 Sep 29;11(9):e0163452. doi: 10.1371/journal.pone.0163452 (PMC5042505; doi:10.1371/journal.pone.0163452)
Supplement: S5 Table — (DOCX) [file pone.0163452.s010.docx]

**S5 Table. The walking plane of the renal artery and renal vein in front of the spine.**

Table 5. The walking plane of the renal artery and renal vein in front of the spine

| Walking plane | Renal artery | | | Renal vein | | |
| --- | --- | --- | --- | --- | --- | --- |
|  | Male (n=30) | Female (n=30) | Male+Female | Male (n=30) | Female(n=30) | Male+Female |
| T12 down 1/3 |  |  |  |  | 1 (3.3%) | 1 (1.7%) |
| L1 up 1/3 |  | 4 13.3%) | 4 (6.7%) | 1 (3.3%) | 1 (3.3%) | 2 (3.3%) |
| L1 middle 1/3 | 7 (23.3%) | 5 (16.7%) | 12 (20.0%) | 1 (3.3%) | 3 (10.0%) | 4 (6.7%) |
| L1 down 1/3 | 6 (20.0%) | 5 (16.7%) | 11 (18.3%) | 7 (23.3%) | 7 (23.3%) | 14 (23.3%) |
| L1/2 intervertebral space* | 5 (16.7%) | 11 (36.7%) | 16 (26.7%) | 6 (20.0%) | 10 (33.3%) | 16 (26.7%) |
| L2 up 1/3 | 8 (26.7%) | 4 (13.3%) | 12 (20.0%) | 9 (30.0%) | 6 (20.0%) | 15 (25.0%) |
| L2 middle 1/3 | 3 (10.0%) | 1 (3.3%) | 4 (6.7%) | 5 (16.7%) | 2 (6.7%) | 7 (11.7%) |
| L2/3 intervertebral space | 1 (3.3%) |  | 1 (1.7%) | 1 (3.3%) |  | 1 (1.7%) |

* The renal artery and renal vein were overlapping in front of the L1/2 intervertebral space in 11 cases (18.3%).
