# Supplementary material for: Imaging Anatomical Research on the Operative Windows of Oblique Lumbar Interbody Fusion
Source: PLoS One. 2016 Sep 29;11(9):e0163452. doi: 10.1371/journal.pone.0163452 (PMC5042505; doi:10.1371/journal.pone.0163452)
Supplement: S6 Table — (DOCX) [file pone.0163452.s011.docx]

**S6 Table. Renal artery and renal vein positioning.**

Table 6. Renal artery and renal vein positioning

|  | Male (n=30) | Female (n=30) | Male+Female |
| --- | --- | --- | --- |
| Renal artery overlapping renal vein | 16 | 15 | 31 (51.7%) |
| Renal artery above, renal vein below | 9 | 11 | 20 (33.3%) |
| Renal vein above, renal artery below | 5 | 4 | 9 (15.0%） |
